# Escitalopram Induced Angioedema

**DOI:** 10.7759/cureus.31600

**Published:** 2022-11-17

**Authors:** Samantha L Conde, Hani Nazha, Cassandra Simpkins

**Affiliations:** 1 Psychiatry and Behavioral Sciences, West Virginia University Health Sciences-Charleston Division/Charleston Area Medical Center, Charleston, USA; 2 Internal Medicine and Psychiatry, West Virginia University School of Medicine, Charleston, USA; 3 Pharmacy, West Virginia University School of Medicine, Charleston, USA

**Keywords:** consultation liaison psychiatry, addiction psychiatry, drug-induced angioedema, pharmacology, escitalopram

## Abstract

Management of psychiatric disorders in high-risk cardiac patients often requires difficult decision making when it comes to acceptable medication side effects. We present the case report of a 28-year-old female with a history of generalized anxiety disorder (GAD), major depressive disorder (MDD), intravenous heroin use disorder, and prior tricuspid valve replacement who presented to the hospital with signs and symptoms of sepsis. She was found to have corrected QT interval (QTc) prolongation and infective endocarditis with blood cultures positive for *Streptococcus viridans*. Due to QTc prolongation, her home medication of citalopram was discontinued in favor of escitalopram. Within 24 hours of administration, the patient experienced angioedema with periorbital swelling, lip swelling, and urticaria of the face and arms which was resolved with intravenous (IV) diphenhydramine. In this case report, we present what we believe to be the first recorded case of escitalopram-induced angioedema in a patient without a past medical history of hereditary angioedema (HAE), and how pharmacogenomic testing influenced antidepressant medication selection.

## Introduction

There have been well-documented cases of both paroxetine and fluoxetine-associated angioedema but only a single documented case of escitalopram-induced angioedema in a patient with hereditary angioedema (HAE) [1-3]. This case report examines the relationship between escitalopram and angioedema in a patient who was not diagnosed with HAE, as well as the difficulties in antidepressant selection and management in a patient who has decreased drug metabolism due to atypical *CYP450* gene expression.

## Case presentation

A 28-year-old Caucasian female presented to the emergency department with a one-week history of chest pain, intermittent subjective fevers, chills, night sweats, and shortness of breath upon exertion. The patient was febrile at 38.8 C, had a heart rate of 118 bpm, blood pressure of 118/80 mmHg, and respiratory rate of 18 breaths per minute. Her notable past medical history included intravenous drug use (IVDU), endocarditis, bioprosthetic tricuspid valve replacement eight months prior, generalized anxiety disorder (GAD), and major depressive disorder (MDD). She endorsed using intravenous heroin daily for the previous month.

**Figure 1:**
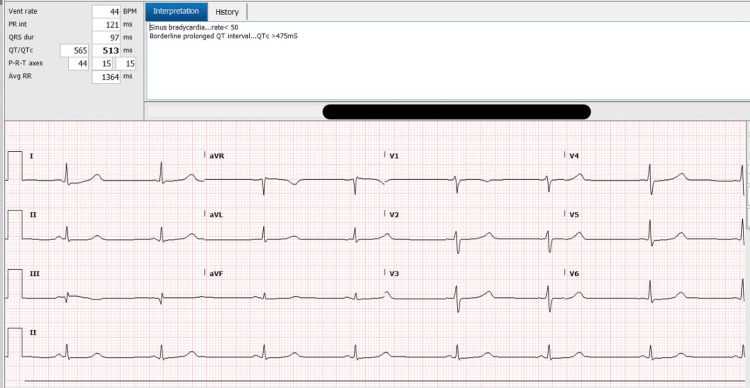
Prolonged QTc interval on patient's electrocardiogram

**Figure 2:**
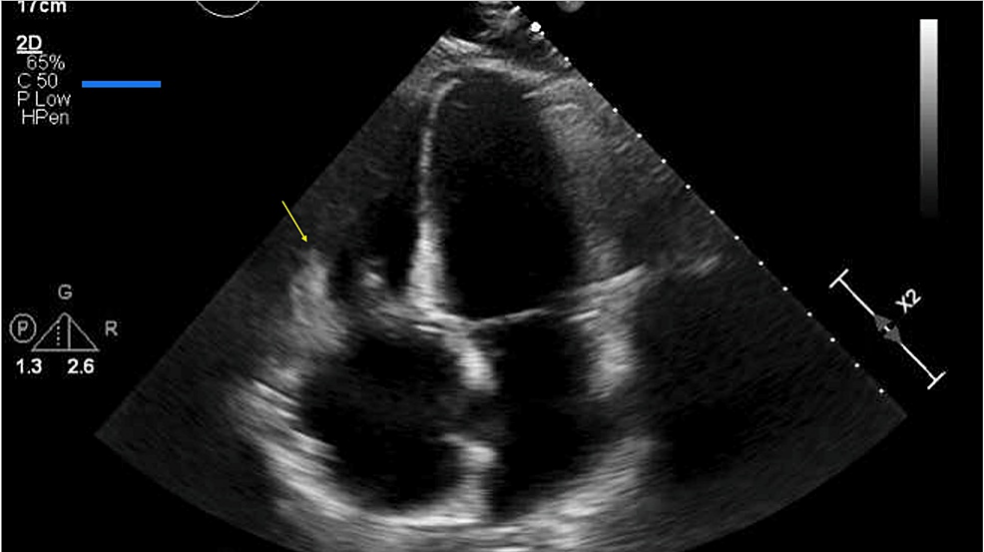
Apical four-chamber view on transthoracic echocardiogram The image is showing medium-sized vegetation on the tricuspid valve.

Laboratory testing was notable for a white blood cell (WBC) count of 23.5 cells/L as well as a troponin-I of 22 ng/L. An electrocardiogram (ECG) revealed sinus tachycardia with a prolonged QTc of 513ms (Figure 1), and a subsequent transthoracic echocardiogram revealed a medium-sized, mobile vegetation on her bioprosthetic tricuspid valve consistent with infective endocarditis (Figure 2). Blood cultures returned positive for *Streptococcus viridans* growth.

Empiric antibiotic treatment for the patient’s infective endocarditis consisted of intravenous (IV) gentamicin 80mg every eight hours and IV ceftriaxone 2g every 24 hours. The patient’s prior diagnosis of MDD and GAD was stabilized with citalopram 40mg daily. However, due to citalopram’s known effect of increasing QTc, this medication was discontinued. Noting the patient’s medical history and the addition of new stressors associated with her current hospitalization, initiation of a different antidepressant was medically necessary. The patient had previously trialed several different selective serotonin reuptake inhibitors (SSRIs), selective serotonin and norepinephrine reuptake inhibitors (SNRIs), and mirtazapine reporting unacceptably severe side effects, among these being worsening mood, drowsiness, and gastrointestinal disturbances. Due to a seizure event on admission day two from suspected benzodiazepine withdrawal, bupropion was not chosen as this medication is known to decrease a patient’s seizure threshold [4].

Escitalopram 5mg daily was chosen as it has been shown to have a smaller effect on QTc prolongation in comparison to citalopram, may reduce the risk of major adverse cardiac events in patients with acute coronary syndrome (ACS), and facilitates abstinence in patients with opioid use disorder [5-8]. Within 24 hours of starting escitalopram, the patient experienced angioedema with periorbital swelling, lip and tongue swelling, urticaria of the face and arms, shortness of breath, and difficulty swallowing. The patient’s symptoms resolved quickly after administering IV diphenhydramine 25mg. In the eight days prior to this event, the patient had been tolerating IV antibiotics without issue and there were no new foods or medications started other than escitalopram. Additionally, her Naranjo Algorithm-Adverse Drug Reaction Probability Scale revealed a total score of five, indicating a probable reaction to escitalopram [9].

Four hours after the resolution of this event, the patient suffered a second, less severe reaction involving urticaria of her left arm and forearm immediately after receiving iopamidol IV contrast during a computerized tomography (CT) scan. Her symptoms quickly resolved after the administration of a second 25mg IV diphenhydramine dose. The patient reported no prior allergic reaction to iopamidol despite having received this IV contrast agent multiple times in the previous two years.

Due to the patient’s history of failed antidepressant trials and her reaction to escitalopram, pharmacogenomic testing was pursued. Of interest was the CYP450 family of enzymes; particularly CYP2C19, CYP2D6, and CYP3A4, which are responsible for the majority of antidepressant metabolism [10]. Testing showed that the patient was considered an intermediate metabolizer with reduced activity of the CYP2D6 and CYP3A4 enzymes. Considering these results, lamotrigine 25mg daily was initiated and the patient was able to tolerate this medication without issue or severe side effects. Additional ECGs performed at one month and 20 months after citalopram discontinuation showed the patient’s QTc to be 441ms and 421ms respectively.

## Discussion

Although the mechanism for this patient’s angioedema is unclear, we theorize that reduced enzymatic metabolism and medication clearance could have increased plasma escitalopram concentration beyond therapeutic levels. Medication-induced serotonin release may have then led to increased vascular permeability and angioedema. An alternative theory credits drug molecules interacting non-covalently with T-cell receptors or major histocompatibility molecules (MHC) to activate the immune system and steer inflammatory cells toward the dermis. This theory was described by Tuman et al. [2] as a potential mechanism for urticaria and angioedema associated with fluoxetine in a similar case report [11].

Considering many antidepressant medications are metabolized hepatically by CYP2C19, CYP2D6, and CYP2C9. Previous drug trial starting doses may have produced higher than intended plasma levels of the medications which could explain why this patient reported such severe side effects [9]. When considering that escitalopram metabolism is shared relatively evenly between CYP2C19 (36%), CYP2D6 (30%), and CYP3A4 (34%), having reduced enzymatic activity in both CYP2D6 and CYP3A4 could theoretically compound the reduction in drug metabolism and increase side effects [12].

While the Clinical Pharmacogenetics Implementation Consortium (CPIC) issued guidelines in 2015 for SSRI administration in patients with CYP2C19 and CYP2D6 polymorphisms [13], we feel that more research is warranted regarding SSRI pharmacokinetics in patients with reduced CYP3A4 enzyme activity. Additionally, pharmacogenomic testing may prove beneficial in patients who report severe side effects after multiple failed antidepressant trials.

## Conclusions

Presented in this case report is a patient who experienced angioedema and urticaria within 24 hours of her first escitalopram dose. Escitalopram-induced angioedema has been previously described in a patient with HAE, but we believe this to be the first case report in a patient who is not diagnosed with HAE. Similar case reports have also been described in patients taking fluoxetine and paroxetine at therapeutic dosages. Further research is required to understand the mechanism behind these occurrences. This case report also demonstrated the difficulty of choosing an antidepressant in a patient with co-morbid medical conditions and the benefit of using pharmacogenomic testing to advance the therapeutic alliance further. 
